# A Half-Century of Anæsthesia

**Published:** 1894-05

**Authors:** 


### Editorial.

### A HALF-CENTURY OF ANÆSTHESIA.

So accustomed have we become to the thought of relief from pain in surgical operations, that very few of the present generation can realize that the immunity from suffering has been introduced within the memory of many now active upon the world's stage. The present century, nearing its close, will go down into history as the period of the greatest achievements, not only in science, mechanics, education, and art, but in the amelioration of human suffering. Through anæsthesia and the discoveries in bacteriology the range of surgical operations has been extended beyond the wildest dreams of the operators of the first half of the present century.

The mind as it contemplates the tortures of the past can but regard the introduction of anæsthesia as one of the greatest blessings ever given to the human family, yet it is surmised that the world moves on its way unconscious of the debt due to those who presented this boon to humanity.

Anæsthesia cannot be classed with the inventions of this prolific age, but rather with its inspirations. The heedless observer would say it was a happy thought when Wells, in 1844, decided to have a tooth removed with nitrous oxide, but an idea of that character would not have entered his active brain except there had been an earnest seeking for an effective means to accomplish the result of extraction without pain. The soil must be prepared both in the mental and physical life before germination can take place, whether that be in a thought or a seed. Hence, judging from this well-known law of development, Dr. Wells was prepared to accept a suggestion looking towards this end when, on December 11, 1844, he sat in the chair to test upon his own person the possibility of removing a tooth without suffering, as severe a test to which probably he could have subjected himself.

The importance of having some means to alleviate pain had not been appreciated by the medical faculty from the beginning of the practice of the healing art, and no systematic measures had been taken to demonstrate the possibility of its accomplishment. So thoroughly was this body imbued with the idea that suffering was a part of the inheritance of the race, that not only was no attempt made to change this condition, but apparently it was not desired. The evidence of this was manifested by the coldness and evident want of interest with which the subject and the original workers were received.

It was, therefore, not surprising that Wells not only met with opposition from the medical faculty, but experienced the fate of all original thinkers of having a host of antagonizing critics, as well as imitators and claimants for the honor of discovery, when it became an honor. That he finally succumbed under the strain is not surprising.

This is not the place to enter into the merits of the controversy in regard to the question as to the discovery of anæsthesia, which has continued until the present time, but a candid and judicial examination of all claims to priority have failed, in the opinion of the writer, to disturb the settled conviction of the dental profession that the Paris Medical Society was justified, in January, 1848, in proclaiming to the world that to Dr. Horace Wells was "due all the honor of having first discovered and successfully applied the use of vapors or gases whereby surgical operations could be performed without pain." The exhaustive "Inquiry into the Origin of Modern Anæsthesia," by Hon. Truman Smith, published in 1867, should be sufficient, we think, to establish this point beyond controversy in all candid minds.

It is natural, perhaps, that medicine has found it a disagreeable memory that this most important aid in the relief of human suffering *should have been the work of a dentist*, and that earnest efforts should have been made to place the honor upon a medical graduate. That this has failed needs only passing reference, and it still remains to the honor of dentistry that it presented to the world this great boon.

Fifty years have passed since that memorable period, and we have now entered upon the year 1894 and can calmly view the work of the past, and, setting aside the acrimonious feelings engendered in 1844, can feel that the time has arrived when we, as a profession, can publicly claim the honor and glorify the event.

In view of these facts the Odontological Society of Pennsylvania proposes, before the close of the present year, to appropriately celebrate this discovery, and it seems to us this very proper initial movement should be regarded as an excellent example to be followed by the dental profession throughout the world. It is not merely essential to demand for dentistry the honor of this work, for that is a minor matter, but it is important as showing to an incredulous world the value of the labor of this body and also the power which concentrated effort possesses when applied to the diminution of human ills in any direction, and that dentistry has demonstrated in its work that it has occupied no minor place in this labor.

A concurrent demonstration of all the dental organizations would certainly be impressive. It would show beyond criticism that professions are not ungrateful, but that they are capable of holding in undying memory their greatest benefactors, and among these Dr. Horace Wells, late of Hartford, Conn., holds the most illustrious place.

